# Photo-Actuation of Liquid Crystalline Elastomer Materials Doped with Visible Absorber Dyes under Quasi-Daylight

**DOI:** 10.3390/polym12010054

**Published:** 2019-12-31

**Authors:** Ban Qin, Wenlong Yang, Jiaojiao Xu, Xiuxiu Wang, Xiangman Li, Chensha Li, Yachen Gao, Qiao-e Wang

**Affiliations:** 1 Key Laboratory of Functional Inorganic Material Chemistry, Ministry of Education of the People’s Republic of China, Heilongjiang University, Harbin 150080, China; BruceChum941006@163.com (B.Q.); X1143790344@163.com (J.X.); wangxiuxiu053@163.com (X.W.); 2 Department of Applied Science, Harbin University of Science and Technology, Harbin 150080, China; yangwenlong1983@163.com; 3 Women and Children Health Centre of Xiangfang District, Harbin 150040, China; huwentaolxm@163.com; 4 Key Laboratory of Electronics Engineering, College of Heilongjiang Province, Heilongjiang University, Harbin 150080, China; gaoyachen@hlju.edu.cn; 5 Key Laboratory of Cosmetic, China National Light Industry, Beijing Technology and Business University, Beijing 100048, China

**Keywords:** liquid crystalline elastomer, visible absorber dyes, quasi-daylight, photothermal conversion, photo-actuation

## Abstract

We studied the effect of visible absorber dyes on the photo-actuation performances of liquid crystalline elastomer (LCE) materials under quasi-daylight irradiation. The dye-doped LCE materials were prepared through infiltrating visible absorber dyes into a polysiloxane-based LCE matrix based on its solvent-swollen characteristic. They demonstrated well absorption properties in visible spectrum range and performed strong actuation upon the irradiation from quasi-daylight source, thus indicating that the presence of visible absorber dyes effectively sensitized the LCE materials to light irradiation since the light energy was absorbed by the dyes and then converted into heat to trigger the phase change of LCE matrix. The photo-actuation properties of dye-doped LCE materials with different visible absorber dyes, varied dye contents, and irradiation intensities were investigated. It was shown that the visible absorber dyes with different absorption bands created different photo-actuation performances of LCE materials, the one whose absorption band is near the intensity peak position of quasi-daylight spectrum created the optimum photo-actuation performance. The result disclosed a valuable light utilization way for photo-controlled LCE materials since it revealed that a light-absorbing dye, whose absorption band is in the high intensity region of light spectrum, is capable of effectively utilizing light energy to drive the actuation of LCE materials.

## 1. Introduction

Liquid crystal elastomer (LCE) materials, as a type of fascinating smart polymer deformable materials, have been intensively studied since Finkelmann and co-workers reported in the 1990s [1,2,3,4,5]. They are formed by linking liquid crystal (LC) mesogenic segments with covalently crosslinked polymer chains. The coupling between the LC orientational order and the elasticity of polymer networks can bring large and reversible anisotropic shape changes of LCE materials under the applied external stimuli [6]. By depending on the structures and compositions, LCE materials can experience shape changes in response to specific external stimuli including magnetism [7,8], heat [9], electric field [10], light [11], ions [12], moisture [13,14], and solvent [15], etc. The outstanding stimuli-responsitivities enable them to be widely used in smart actuators [16,17,18,19,20,21], biomimetic devices [22,23,24,25], artificial organs [26,27,28], and microrobots [29,30,31], etc.

**Scheme 1:**
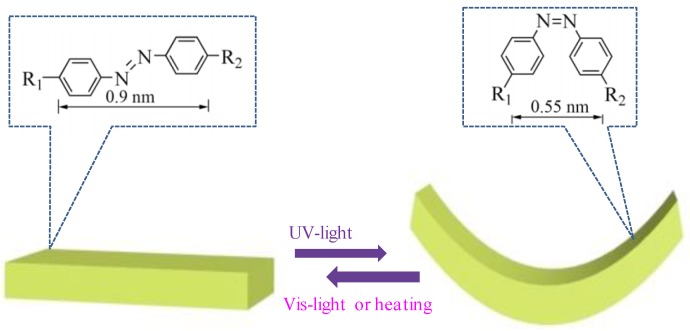
Illustration of light driven deformation of azobenzene contained liquid crystalline elastomer (LCE) materials.

Thermal stimuli and light stimuli play major roles in various applications of LCE materials [4,11]. However, thermal stimuli may not be an ideal method since it generally needs heating apparatus to contact the materials which act as the actuating components in mechanical devices. Light stimuli may be more desirable because of the superiorities of remote and precise controlling without any physical contact, in a non-damaging manner, rapid switching on/off, and clean energy sources. The classical light stimuli responsive LCE materials were developed by incorporating photochromic molecule systems capable of generating photodeformation, such as azobenzene chromophores, into the polymer backbones of crosslinked LC networks [2,5], the chromophores can undergo light-induced trans-cis isomerization, which disrupts the LC phase and hence drives the deformations of LCE materials [2,5], as shown in Scheme 1. There is recently an emergence on using photo-thermo-mechanical effect to expand the application of photo-actuation in LCE materials. Such LCE materials are generally fabricated through the incorporation of light-absorbing components with LCE matrices, the light-absorbing components absorb and convert light energy into heat which then drives the deformations of LCE materials [9,32]. Carbon nanotubes [33,34,35,36,37,38,39], graphene materials [40,41,42,43], noble metallic nanoparticles [44,45,46,47,48,49,50], and conjugated polymer particles [51] have been proved to be the effective components of photothermal conversion. However, the heterogeneity because of hetero-phase structure and agglomeration of particles can hamper the performances of LCE materials. To overcome this problem, Marshall and Terentjev first utilized light-absorbing organic dyes as the components of photothermal conversion to realize the photo-actuation of LCE materials for reason that they could be dispersed into LCE matrices in molecular sizes [52]. From then on, many near-infrared (NIR) responsive dye-doped LCE materials have been developed [25,40,53,54,55,56,57,58].

Quasi-daylight sources, with the emission spectra being similar to nature sun-light, generally possess the advantages of economy, convenience, and health, thus it would be more valuable to effectively utilize the energy of quasi-daylight, even the nature sunlight, to drive the actuation of LCE materials. In this work, three visible absorber dyes, whose absorption bands are in the range of visible spectrum, were used to develop the dye-doped LCE materials which can be actuated by quasi-daylight, and a classical polysiloxane side-chain LCE matrix was used as the host material.

The dye-doped LCE materials are conventionally prepared via in situ synthesis by adding the dye molecules into synthetic reaction mixtures [25,40,52,54,55,57]. But the dye molecules may influence the synthesis reactions, while the phase separation may be induced by the synthesis reactions, resulting in the aggregation of dye molecules [59]. Some dye-doped LCE materials can be fabricated by the way of chemical bonding light-absorbing chromophore units into LCE networks [53,56]. But this would distinctly increase the difficulty and complexity of material designs and fabrications. In our work, by utilizing the swelling characteristic of LCE networks in certain solvents [6,11], the dye-doped LCE materials were prepared through infiltrating the dye molecules into the LCE matrix with the solvent, thus avoiding the side chemical changes during the preparation and ensured the well dispersibility of the dye molecules in LCE matrix. The prepared dye-doped LCE materials exhibited basically coincident photo absorption properties as the corresponding pure visible absorber dyes in quasi-daylight region. Benefiting from the light-absorbing and photothermal conversion effects of the doped visible absorber dyes, strong photo-driven deformation performances of the dye-doped LCE materials were obtained under the irradiation of quasi-daylight, and the photo-actuation behaviors were fully reversible after the light irradiation was removed. The photo-actuation properties of every dye-doped LCE materials depended on light intensity and dye content. However, the photo-actuation performances of the LCE materials doped with different visible absorber dye were different, the one whose absorption band is near the intensity peak position of quasi-daylight spectrum demonstrated the best photo-actuation properties at every light intensity and dye content. The results indicated that the light-absorbing dyes with the absorption bands being in the high intensity region of light spectrum can more effectively utilize the light energy to drive the actuation of LCE materials, and thus were significant for the improvement of light energy utilization of photo-controlled LCE materials, while paving way for further development of the sun-light actuated LCE actuators.

## 2. Experimental

### 2.1. Preparation of Materials

The mesogenic monomer was 4-methoxyphenyl 4-(3-butenyloxy)benzoate (MBB), the cross-linker was 1,4-*bis*(undec-10-enyloxy)benzene (11UB), and the catalyst solution was dichloro (1, 5-cyclooctadiene) platinum (II) (Pt (COD) Cl_2_) dissolved in toluene. They were synthesized or prepared by following our previous work [50]. The polymer backbone, which is poly-dimethylhydrosiloxane (PMHS) with the molecular weight range from 1800 to 3200 Dalton, was obtained from Aldrich (St Louis, MO, USA). The visible absorber dyes (ABS407, ABS594 and ABS694) were purchased from Luxottica Exciton (Lockbourne, OH, USA).

**Scheme 2:**
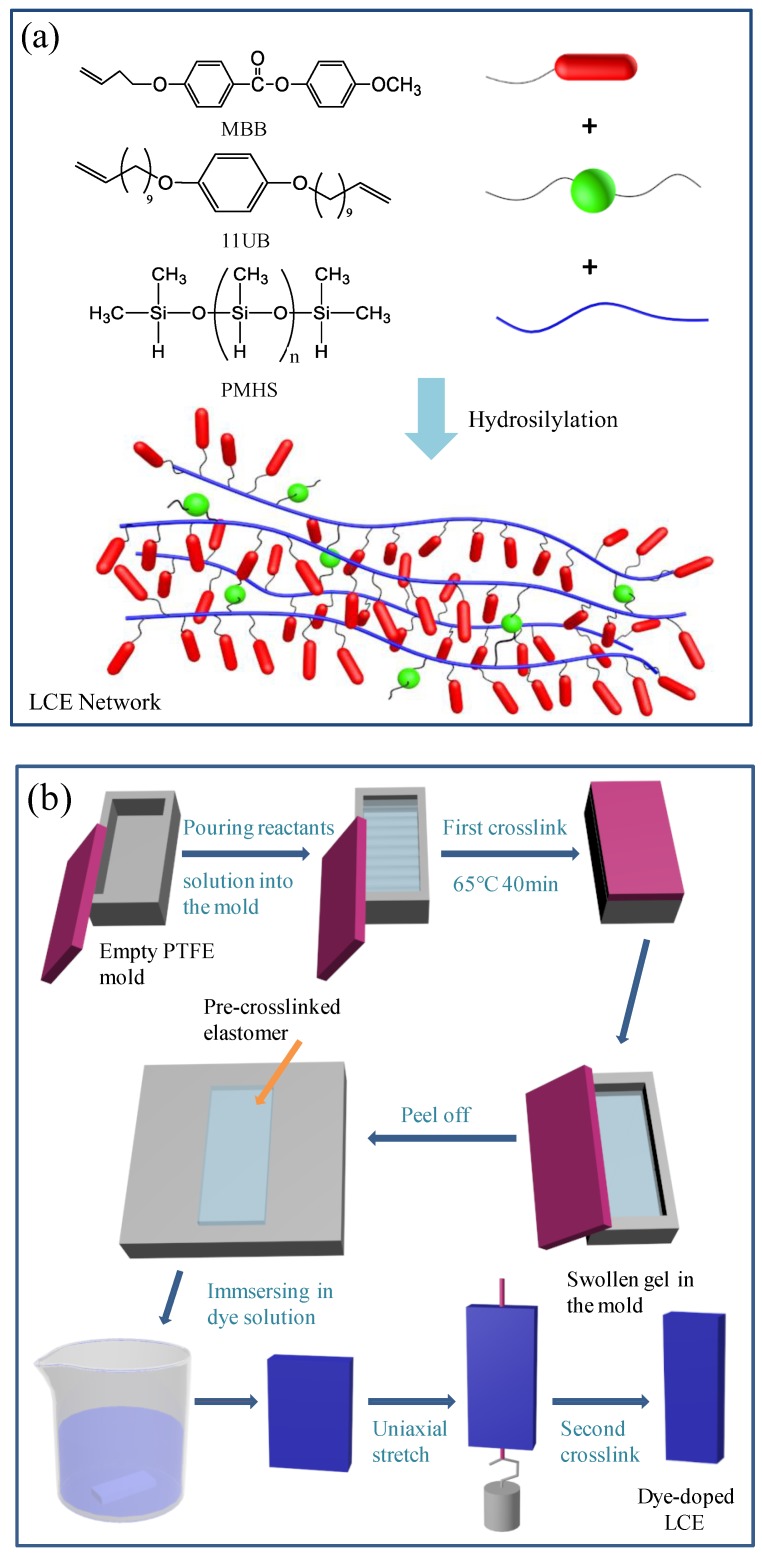
(**a**) The molecular structures of reactants and the synthesis of LCE network; (**b**) Illustration of the preparation of dye-doped LCE materials.

The synthesis of the side-chain LCE network with polysiloxane backbone was via a sol–gel process of hydrosilation reaction which achieved an effective 15% cross-linking density, as shown in Scheme 2a. The measurement of attenuated total reflection Fourier transform infrared spectroscopy (ATR-FTIR) spectrum of the polysiloxane side-chain LCE confirmed the generation of LCE networks through hydrosilation crosslinking, as illustrated in Figure S1 and Support Information (S-I). The preparation of polysiloxane side-chain LCE materials doped with visible absorber dyes was based on the way of classical two-step crosslinking coupled with a stretching process, the experiment protocol was similar to our previous work [50] but some detailed procedures were altered. As shown in Scheme 2b, a precursor reactant mixture composed of 0.048 g PHMS, 0.184 g MBB (0.62 mmol), 0.0227 g 11UB (0.055 mmol), and proper amount of Pt(COD)Cl_2_ catalyst solution dissolved in 0.8 mL of toluene was filled into a Teflon mold, and then underwent the first step crosslinking in an oven at 65 °C for 40 min to generate a partially crosslinked toluene-swollen gel elastomer. After the contained toluene was fully evaporated through drying in air, the de-swollen elastomer was immersed in 0.2, 0.4, or 1.2 mL of dye-dissolved toluene solution with a visible absorber dye concentration of 7.5 mg/mL for about 10 min, 20 min, or 40 min. When the dye-dissolved toluene solution was fully absorbed by the elastomer, the re-swollen elastomer was dried again for 40 min to let the contained toluene fully evaporate while the dye molecules retained. The dye containing elastomer was uniaxially stretched under a load to attain a stable length and form a monodomain structure, and then heated in the oven at 70 °C overnight to complete the second step of crosslinking and stabilize the monodomain structure. Through the above procedures, dye-doped nematic LCE materials with dimensions of 40 mm × 10 mm × 0.6 mm, and mass content of visible absorber dye of 0.1 wt %, 0.3 wt %, or 0.6 wt % according to composition ratios, were respectively prepared. A blank nematic LCE material without dye doping was prepared by the same way but without the procedure of infiltrating dye molecules into the LCE matrix. Herein, the first crosslinking step was critical and the reaction condition for it was optimal in our work. The preparation of LCEs would fail if the reaction time of the first crosslinking step is too short or too long, as expatiated in the Support Information (S-II) and demonstrated in Figure S2.

### 2.2. Characterization Methods

**Scheme 3:**
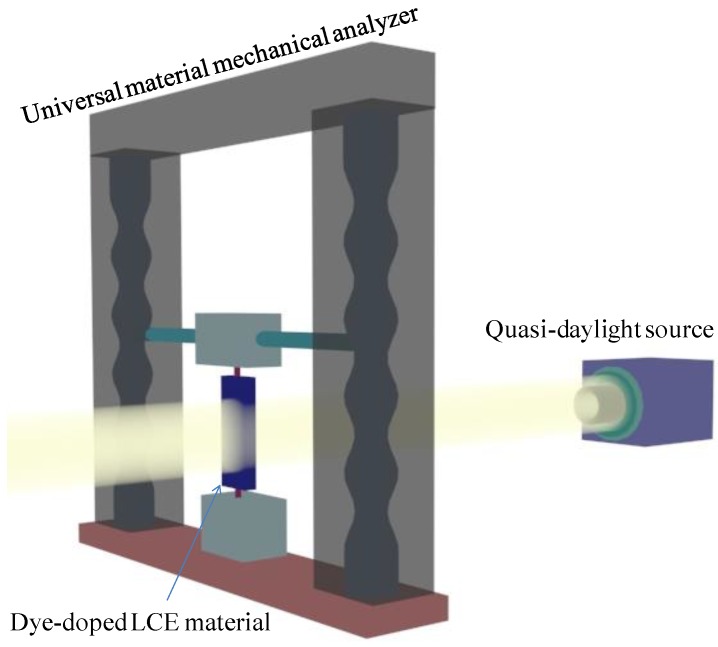
Illustration of the experimental setup for measuring the photo-stimulated axial strains of LCE materials by the universal material mechanical analyzer.

The mesomorphic and phase transformation properties of LCE materials were investigated by polarizing optical microscopy (POM, Nikon Instruments, SMZ 1500, Melville, NY, USA) observations and differential scanning calorimetry (DSC, TA Instruments Q100 modulated differential scanning calorimeter, New Castle, DE, USA) measurements, respectively. The UV-vis-NIR absorption spectra of the dyes and dye-doped LCE materials were measured by using a USB4000 spectrometer. The photo-responsive actuations of LCE materials were performed by using a quasi-daylight source (New Port, Oriel Sol3A, 210W power, Irvine, CA, USA). As shown in Scheme 3, the strain changes of LCE materials in response to light stimulus were measured in situ on a universal material mechanical analyzer (CMT-10, LG Company, Jinan, China). The two ends of the tested material sample were gripped by the pair tension clamps assembled on the mechanical analyzer, and the pre-applied tensile stress was 60 kPa. An illuminance measuring instrument (FLUKE 941, Avery De, USA) was used to measure the light irradiation intensity which could be tuned by turning the knob assembled on the light source, and the temperature changes of material samples upon irradiation were measured in situ by using a multilogger thermometer (HH 506 RA, OMEGA Engineering, Stamford, CT, USA). The environment temperature of experiments was 25 °C.

## 3. Results and Discussion

**Figure 1:**
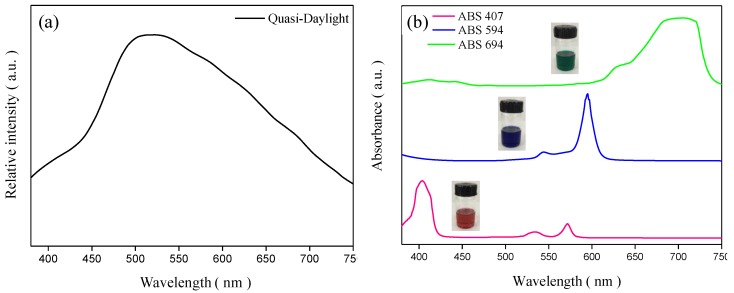
(**a**) The light spectrum of quasi-daylight; (**b**) the UV-vis-NIR absorption spectra of visible absorber dyes dissolved in toluene (7.5 mg/mL), the inserted images are the photos of corresponding toluene solutions of ABS407, ABS594, and ABS694 with 7.5 mg/mL of dye concentration.

Suitable optical wavelength absorptions of dyes are needed in order to achieve the photo-actuation of dye-doped LCE materials. Figure 1 shows the light spectrum of our quasi-daylight source, the photo image and UV-vis-NIR absorption spectrum of every visible absorber dye dissolved in toluene. The toluene solutions of ABS407, ABS594, and ABS694 were transparent colored solutions which exhibited a strong absorption in the visible optical wavelength ranges of about 380–420 nm, 580–610 nm, and 670–720 nm respectively, as shown in Figure 1b. By comparing Figure 1a,b, it shows that the above main absorption bands of the three visible absorber dyes are in the region of quasi-daylight spectrum. Thus they are suitably applied in developing the dye-doped LCE materials which can be actuated by quasi-daylight.

**Figure 2:**
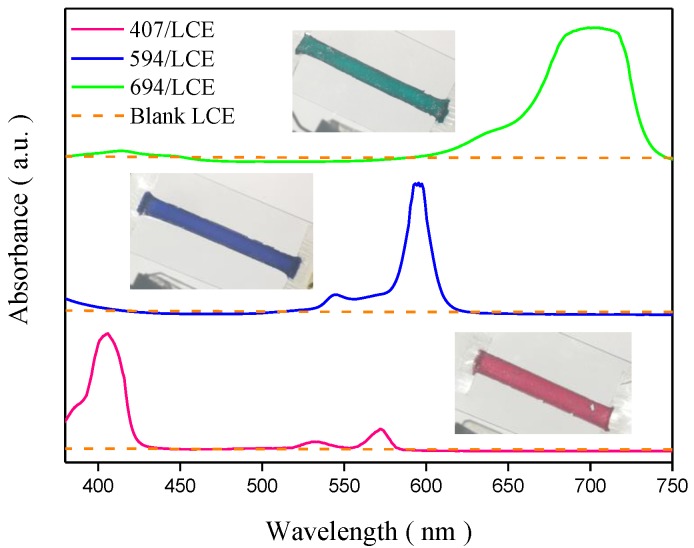
The UV-vis-NIR absorption spectra of the blank LCE and dye-doped LCE materials with 0.6 wt % of visible absorber dyes content. The inserted images are the photos of corresponding 407/LCE, 594/LCE, and 694/LCE with 0.6 wt % of visible absorber dyes content.

Though our dye-doped LCE materials might be fabricated via in situ synthesis since the used visible absorber dyes are toluene soluble and thus can be dissolved in the reaction solution. It should be noted that the dye molecules may influence the synthesis reaction, leading to poor material quality, if directly mixing them into the reaction mixture solution, while the phase separation may be induced by the synthesis reactions in solutions, resulting in the tiny aggregations of dye molecules [59]. In our work, by utilizing the toluene-swollen characteristic and the classical two-step crosslinking process of polysiloxane-based LCE matrices, the dye molecules dissolved in toluene were infiltrated into the LCE matrix with toluene solvent after the first crosslinking step, and then the dye-doped LCE materials were obtained through the stretching process and second crosslinking step, as illustrated in Scheme 2b. Figure 2 shows the photo images of the three kinds of dye-doped LCE material and the UV-vis-NIR absorption spectra of the blank LCE and dye-doped LCE materials, it indicates that every dye-doped LCE material had a uniform color which was consistent with the corresponding visible absorber dye dissolved in toluene, as shown in Figure 1b. In addition, by comparing Figure 2 with Figure 1b, the 407/LCE, 594/LCE, and 694/LCE respectively exhibited an approximate absorption spectrum and consistent main absorption band with the ABS407, ABS594, and ABS694 respectively, while the blank LCE showed no absorption feature. These results revealed that the dye molecules were homogeneously dispersed in LCE matrix with no chemical changes.

**Figure 3:**
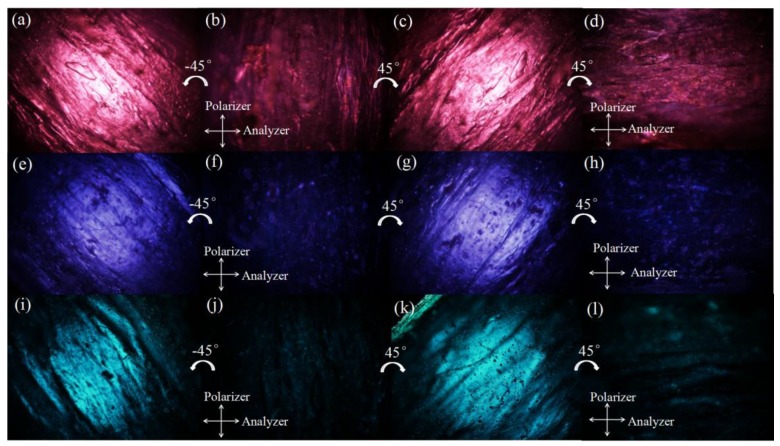
Polarizing optical microscopy (POM) of 407/LCE (**a**–**d**), 594/LCE (**e**–**h**), and 694/LCE (**i**–**l**) with 0.6 wt % of visible absorber dye content. Inserted cross arrows illustrate the polarization directions of the polarizer and analyzer; (**a**,**e**,**i**) the angle between the stretch directions of the dye-doped LCE materials and the polarization directions is −45°; (**b**,**f**,**j**) the stretch directions of the dye-doped LCE materials are parallel to the polarizer; (**c**,**g**,**k**) the angle between the stretch directions of the dye-doped LCE materials and the polarization direction is 45°; (**d**,**h**,**l**) the stretch directions of the dye-doped LCE materials are parallel to the analyzer.

Figure 3, the POM images of three kind dye-doped LCE materials with 0.6 wt % of dye content shows the observed transmittances of a probe light through the crossed polarizer and analyzer with material sample between them. The highest transmittance appeared when the polarizer or analyzer tilted at an angle of ±45 deg to the stretch direction of every material sample, while full birefringence extinction could be observed when the stretch direction was perpendicular or parallel to the polarizer or analyzer. Periodic changes of bright and dark images were observed by rotating every LCE material sample with an interval of 45°. The POM observations of the blank LCE and the 407/LCE, 594/LCE, and 694/LCE containing different content of visible absorber dyes exhibited consistent result. The POM measurements evidenced that the prepared LCE materials had a monodomain nematic phase structure with uniaxial alignment of mesogens, which was well along the stretch direction.

**Figure 4:**
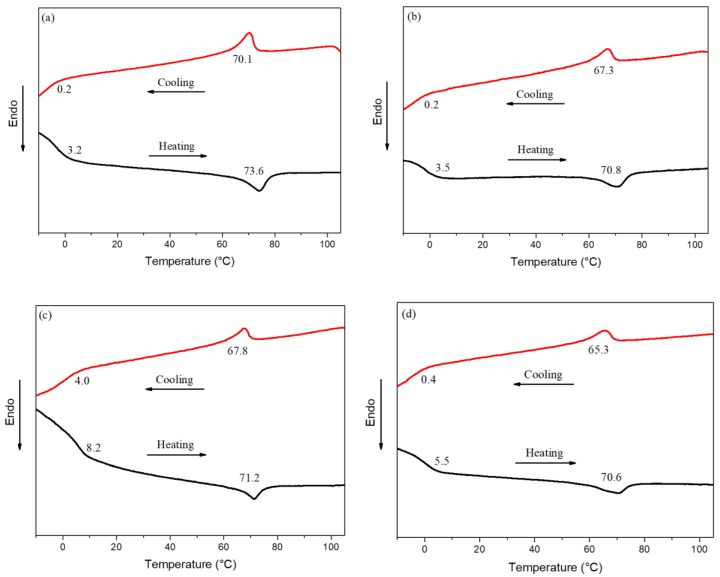
Differential scanning calorimetry (DSC) data curves of heating (black curves)/cooling (red curves) scans at a rate of 5 K min^−1^ of the blank LCE (**a**) and dye-doped LCE materials with 0.6 wt % of visible absorber dye content (**b**–**d**). (**b**) 407/LCE; (**c**) 594/LCE; (**d**) 694/LCE.

The DSC measurement data curves of the prepared blank LCE and three kinds of dye-doped LCE material are shown in Figure 4. The heating scans indicate that their glass transition temperatures (*T*_g_) were in the range of about 3–8 °C, and their nematic-isotropic transition temperatures (*T*_ni_) were in the range of about 70–73 °C. The LCE materials all had an enantiotropic nematic phase. Because of the very low doping ratios (0.6 wt %) of the dyes, the *T*_ni_ of the dye-doped LCE materials were close to that of the blank LCE with a small deviation of below 3 °C.

**Figure 5:**
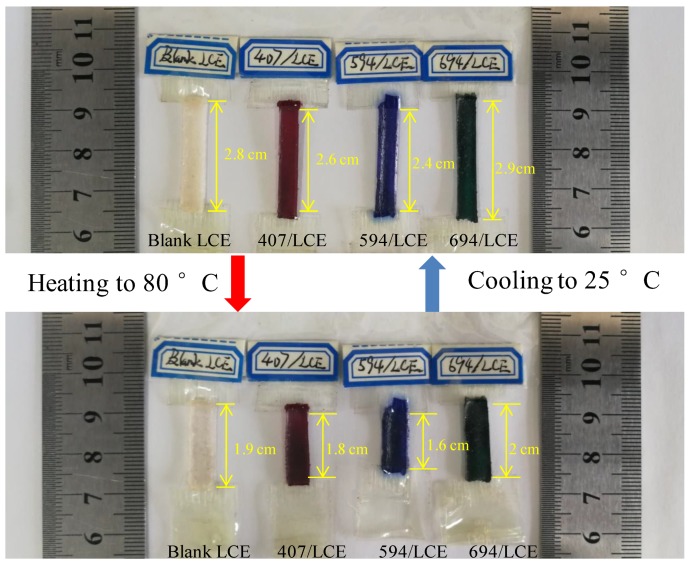
Photo images of thermo-actuations of blank LCE and dye-doped LCE materials with 0.6 wt % of visible absorber dye content on a hot stage.

The prepared blank LCE material and dye-doped LCE materials demonstrated inherent thermo-actuation performance. As shown in Figure 5, they performed reversible axial contraction and restoration along the alignment directions upon the heating/cooling cycles, the maximum axial contractions when heating above the *T*_ni_ were about one third of their original lengths. The mechanism lied in the temperature dependence of mesogenic order. When monodomain LCE materials are heated above the *T*_ni_, the mesogenic orders decrease and the LCE networks become isotropic state, hence the LCE materials show contraction along the alignment directions. When the temperature decrease below the *T*_ni_, the mesogenic orders increase and the LCE networks revert to nematic structure, hence the LCE materials experience an expending process and restore to the initial dimensions [9].

**Figure 6:**
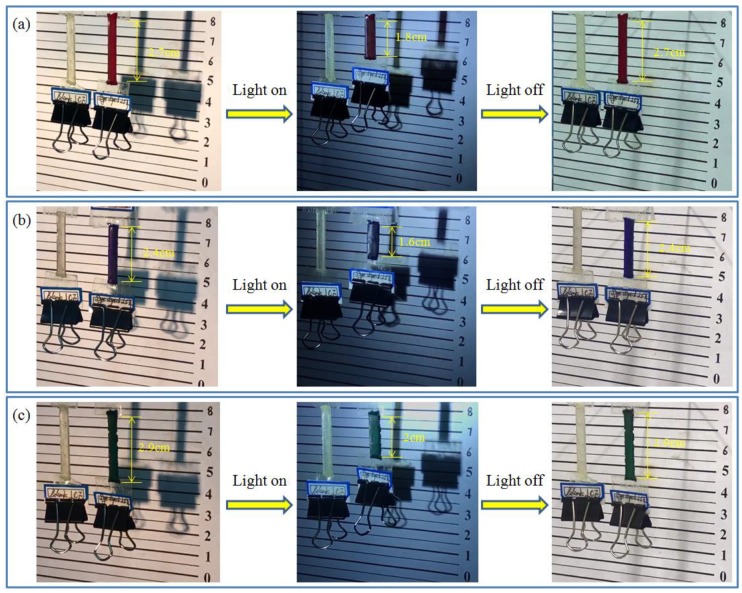
Photo images of photo-actuations of blank LCE and dye-doped LCE materials with 0.6 wt % of visible absorber dye content under quasi-daylight irradiation: (**a**) A pair of blank LCE and 407/LCE; (**b**) a pair of blank LCE and 594/LCE; (**c**) a pair of blank LCE and 694/LCE. The two LCE materials of every pair are hanged side by side, and each sample is loaded by 3 g of weight. The irradiation intensity here is 5.0 × 10^5^ lux.

The dye-doped LCE materials also demonstrated attractive photo-driven deformation behaviors. Figure 6 shows the responses of the blank LCE and three kinds of dye-doped LCE material with 0.6 wt % of dye content under the quasi-daylight irradiation of 5.0 × 10^5^ lux in intensity. When being exposed to the incident light irradiation, the dye-doped LCE materials behaved axial contraction and the finally reached maximum contraction amplitudes were about one-third of their original lengths, which were consistent with those under heating. After the light source was switched off, the dye-doped LCE materials restored to their initial lengths, indicating completely reversible photo-actuation. The blank LCE demonstrated almost no discernible response under the same light irradiation. The visible absorber dyes dispersed in LCE matrix could absorb the energy of quasi-daylight irradiation since their main absorption bands are in the region of quasi-daylight spectrum. The absorbed light energy was converted into thermal energy through molecular vibrations, and then the generated thermal energy heated the LCE matrix, resulted in the axial contraction behavior of the dye-doped LCE materials. When the LCE matrix was heated up to the nematic-isotropic phase transition, the axial contraction reached the maximum amplitude. The blank LCE had almost no light absorption ability, and thus was weakly responsive to light irradiation. The photothermal conversion effect of the visible absorber dyes was confirmed by comparing the temperature changes of the dye-dissolved toluene solutions and the pure toluene solvent upon light irradiation, as expatiated in the Support Information (S-III) and illustrated in Figure S3.

**Figure 7:**
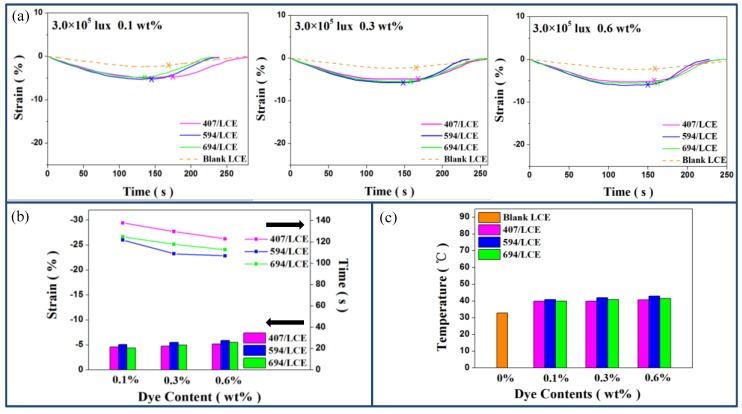
(**a**) Changes in axial strain of blank LCE and dye-doped LCE materials, with 0.1 wt %, 0.3 wt %, and 0.6 wt % of visible absorber dye content respectively, versus time under quasi-daylight irradiation with 3.0 × 10^5^ lux of intensity. The mark “×” at every curve indicates the time when the light source was turned off; (**b**) the final contraction strain (guided by the lower arrow) and the recorded irradiation time for reaching the final contraction strain (guided by the upper arrow) of every dye-doped LCE material under quasi-daylight irradiation with 3.0 × 10^5^ lux of intensity; (**c**) the measured highest temperatures of every LCE material under quasi-daylight irradiations with 3.0 × 10^5^ lux of intensity.

**Figure 8:**
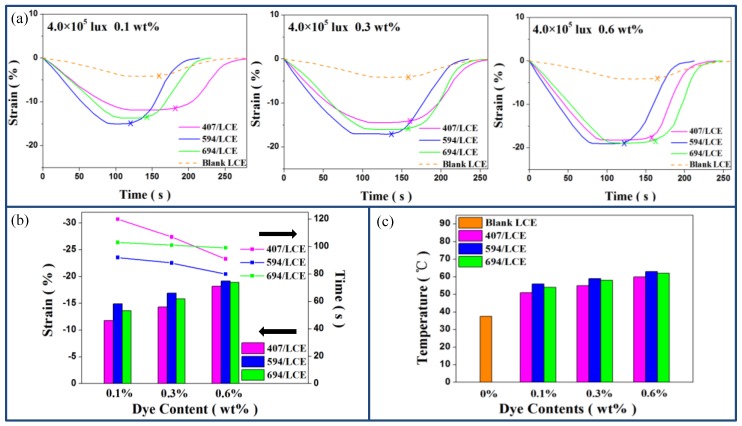
(**a**) Changes in axial strain of blank LCE and dye-doped LCE materials, with 0.1 wt %, 0.3 wt %, and 0.6 wt % of visible absorber dye content respectively, versus time under quasi-daylight irradiation with 4.0 × 10^5^ lux of intensity. The mark “×” at every curve indicates the time when the light source was turned off; (**b**) the final contraction strain (guided by the lower arrow) and the recorded irradiation time for reaching final contraction strain (guided by the upper arrow) of every dye-doped LCE material under quasi-daylight irradiation with 4.0 × 10^5^ lux of intensity; (**c**) the measured highest temperatures of every LCE material under quasi-daylight irradiations with 4.0 × 10^5^ lux of intensity.

**Figure 9:**
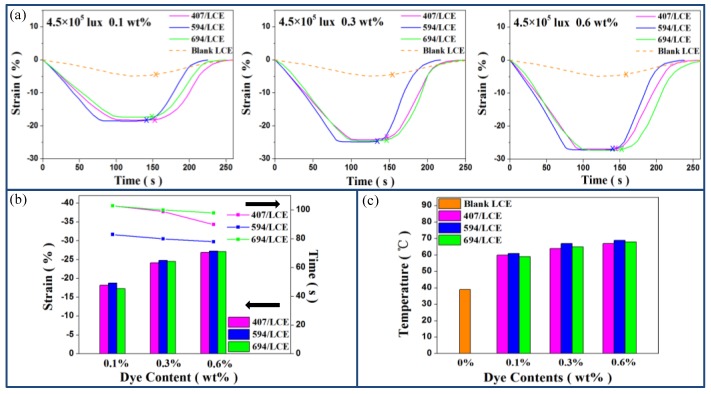
(**a**) Changes in axial strain of blank LCE and dye-doped LCE materials, with 0.1 wt %, 0.3 wt %, and 0.6 wt % of visible absorber dye content respectively, versus time under quasi-daylight irradiation with 4.5 × 10^5^ lux of intensity. The mark “×” at every curve indicates the time when the light source was turned off; (**b**) the final contraction strain (guided by the lower arrow) and the recorded irradiation time for reaching the final contraction strain (guided by the upper arrow) of every dye-doped LCE material under quasi-daylight irradiation with 4.5 × 10^5^ lux of intensity; (**c**) the measured highest temperatures of every LCE material under quasi-daylight irradiations with 4.5 × 10^5^ lux of intensity.

**Figure 10:**
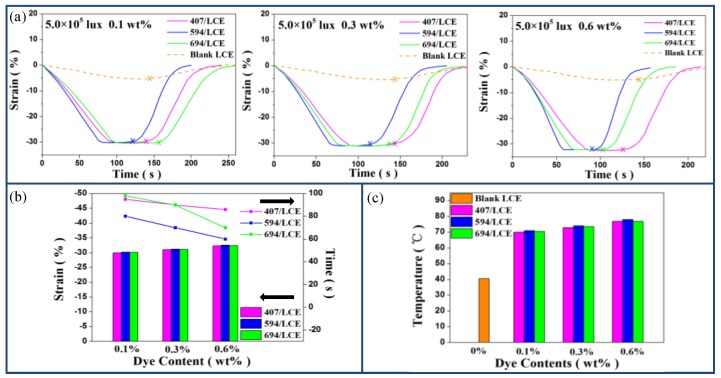
(**a**) Changes in axial strain of blank LCE and dye-doped LCE materials, with 0.1 wt %, 0.3 wt %, and 0.6 wt % of visible absorber dye content respectively, versus time under quasi-daylight irradiation with 5.0 × 10^5^ lux of intensity. The mark “×” at every curve indicates the time when the light source was turned off; (**b**) the final contraction strain (guided by the lower arrow) and the recorded irradiation time for reaching final contraction strain (guided by the upper arrow) of every dye-doped LCE material under quasi-daylight irradiation with 5.0 × 10^5^ lux of intensity; (**c**) the measured highest temperatures of every LCE material under quasi-daylight irradiations with 5.0 × 10^5^ lux of intensity.

The axial strains of the blank LCE and three kinds of dye-doped LCE material with different dye contents, under different irradiation intensities, versus irradiation time, measured by the universal material mechanical analyzer shown in Scheme 3, are plotted in Figure 7a, Figure 8a, Figure 9a and Figure 10a. The contraction strains gradually increased as the irradiation time prolonged in the beginning, but finally became stable for reason that the temperatures of the LCE materials did not continue to rise or the contraction strains of the LCE materials already reached the maximum contraction ratio and thus could not continue to increase with the increased temperature. After the light irradiation was removed, the axial strains of the LCE materials began to decrease and finally attained to the initial zero value. Because of the weak light absorption effect of the blank LCE, its final contraction strain did not exceed 5% under every irradiation intensity, and the achieved highest temperature on it did not exceed 40 °C which was far below its *T*_ni_, as shown in Figure 7c, Figure 8c, Figure 9c and Figure 10c. In addition, it indicates a trend from Figure 7b,c; Figure 8b,c; Figure 9b,c and Figure 10b,c that for every kind of dye-doped LE material, as the dye content increased, the contraction strain rate [60], final contraction strain, and the achieved highest temperature in response to the light irradiation also increased because the increased dye content increased the absorption rate and photothermal conversion rate.

Figure 7b shows that the light irradiation with 3.0 × 10^5^ lux intensity drove very small contraction strains, the final contraction strains of the dye-doped LCE materials were below 6.5% and had no clear difference each other. The achieved highest temperatures in dye-doped LCE materials were below 45 °C and also had no clear difference each other, as shown in Figure 7c.

Figure 8b shows that at an irradiation intensity of 4.0 × 10^5^ lux, the contraction strains of the three kinds of dye-doped LCE material with different dye contents increased but obviously did not reach the maximum contraction ratio since their final contraction strains were below 20%. Figure 8c shows that at this irradiation intensity, the achieved highest temperatures in dye-doped LCE materials also obviously did not attain their *T*_ni_. But among the three kinds of dye-doped LCE material, the strain rate, final contraction strain, and temperature rise under irradiation of the 594/LCE were all higher than those of the 407/LCE and 694/LCE with the same dye content, as shown in Figure 8b,c. The reason should be attributed to the stronger light energy absorption of 594/LCE relative to the 407/LCE and 694/LCE. By comparing Figure 1a and Figure 2, it can be seen that the main absorption band of 594/LCE is near the intensity peak position of quasi-daylight spectrum, while the main absorption bands of 407/LCE and 694/LCE are in the lower intensity regions of quasi-daylight spectrum. Thus the 594/LCE could absorb more energy of quasi-daylight irradiation compared to 407/LCE and 694/LCE, and hence brought a higher temperature rise, strain rate, and contraction strain.

At 4.5 × 10^5^ lux of irradiation intensity, the dye-doped LCE materials still could not reach the maximum contraction ratio, and the highest achieved temperatures under irradiation were still below their *T*_ni_, although their final contraction strains and temperature rises under irradiation were larger than the states under 4.0 × 10^5^ lux of irradiation intensity because of the increase of light energy absorption, as shown in Figure 9b,c. In addition, Figure 9b,c shows that at irradiation intensity of 4.5 × 10^5^ lux, the final contraction strains and temperature rises of the three kinds of dye-doped LCE materials under irradiation began to gradually approach each other as the dye contents increased from 0.1 wt % to 0.6 wt %, but the 594/LCE still demonstrated an obvious higher strain rate compared to 407/LCE and 694/LCE at every dye content because of the increased absorption of quasi-daylight energy.

When irradiation intensity was increased to 5.0 × 10^5^ lux, the final contraction strains of the three kinds of dye-doped LCE materials began approaching each other at every same dye content, and were all above 30%, as shown in Figure 10b. The three kinds of dye-doped LCE materials with 0.6 wt % of dye content reached the maximum contraction ratio since their final contraction strains were above 32%, and the measured highest temperatures under irradiation of them were above 78 °C which was obviously above their *T*_ni_, as shown in Figure 10b,c. However, the 594/LCE still demonstrated the highest strain rate among the three kinds of dye-doped LCE materials at every dye content, as shown in Figure 10b, revealing its best sensitivity of photo-actuation because of the effective utilization of quasi-daylight energy.

In addition, our experiments exhibited that the strain rates of the dye-doped LCE materials in response to light irradiation slightly decreased as the material thickness increased. The reason is that a larger material thickness could cause a higher resistance of thermal diffusion, resulted in a slower photo-actuation rate of LCE material, as expatiated in the Support Information (S-IV) and demonstrated in Figure S4.

**Figure 11:**
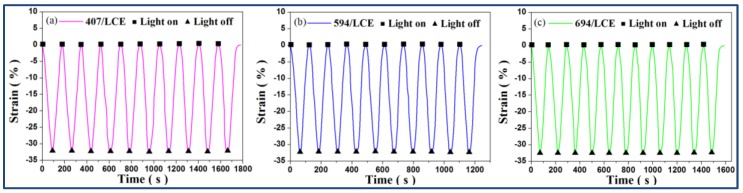
The axial strains of dye-doped LCE materials with 0.6 wt % of visible absorber dye content under repeated switching on/off cycles of quasi-daylight irradiation. (**a**) 407/LCE; (**b**) 594/LCE; (**c**) 694/LCE. The irradiation intensity was 5.0 × 10^5^ lux.

The axial strains of 407/LCE, 594/LCE and 694/LCE with 0.6 wt % of dye content versus irradiation time under repeated on-off switch of quasi-daylight irradiation with 5.0 × 10^5^ lux of intensity are plotted in Figure 11. The three kinds of dye-doped LCE material demonstrated stable reversible deformations and basically consistent maximum contraction ratios during many cycles of on-off switch of quasi-daylight irradiation, revealing their strong anti-fatigue ability in utilization. In addition, Figure 11 indicates that the time taken on 594/LCE was less than that on 407/LCE and 694/LCE for completing the same times of contraction/recovery cycles, revealing the best photo-actuation performance of 594/LCE.

## 4. Conclusions

The utility of photo responsive dyes in LCE matrices involves a novel route of light energy absorption and energy conversion molecular units which provide effective photo-actuation performance to the LCE materials, while incorporating the visible absorber dyes into LCE matrices is an effective way to realize the actuation of LCE materials by using quasi-daylight. By utilizing the toluene-swollen characteristic of polysiloxane-based LCE materials, we prepared three kinds of dye-doped LCE materials through infiltrating the toluene solution of visible absorber dyes into the polysiloxane LCE matrix. The prepared dye-doped LCE materials exhibited basically coincident photo absorption properties as the corresponding pure visible absorber dyes, and demonstrated prominent photo-thermo-mechanical actuation performances. The maximum axial contractions of these dye-doped LCE materials under the irradiation of quasi-daylight were consistent with the situation of thermo-actuation, and the reversible axial contraction/extension deformations could be steadily performed upon the repeated on-off switch of light irradiation. The photo-actuation properties of every kind of dye-doped LCE material depended on the light intensity and dye content. However, the 594/LCE demonstrated better photo-actuation properties compared to the 407/LCE and 694/LCE at every light intensity and dye content. The reason is that the absorption band of the dye molecules contained in 594/LCE is in the high intensity region of quasi-daylight spectrum, thus they can more effectively utilize the energy of quasi-daylight to drive the actuation of LCE material. The research results revealed that the addition of specially selected dyes to LCE materials could offer a useful alternative route for the programming of photo-controlled smart actuators based on LCE materials, and would be of great guiding significance for the effective energy utilization of wide spectrum lights, including the nature sunlight, in the actuation of photo-controlled LCE materials. Many of the cyanine dyes and coordination complex dyes have good light absorption properties and are suitable for the development of dye-doped LCE materials; their properties can be improved or tuned through by constituting coordinative bonds, incorporating functional chromophores or substitute groups, changing the coordinative metal, or constituting different conjugation effect [61,62,63], etc. In the future, we will continue to explore the ways of enhancing the photo-actuation performances of dye-doped LCE materials through studying the correlation of chemical structure and properties of dye molecules correlating to light absorption and photothermal conversion.

## Supplementary Materials

The following are available online at https://www.mdpi.com/2073-4360/12/1/54/s1.
