# Supplementary material for: Photo-Actuation of Liquid Crystalline Elastomer Materials Doped with Visible Absorber Dyes under Quasi-Daylight
Source: Polymers (Basel). 2019 Dec 31;12(1):54. doi: 10.3390/polym12010054 (PMC7023533; doi:10.3390/polym12010054)
Supplement: Supplementary file 1 [file polymers-12-00054-s001.pdf]

polymers

MDPI

# Support information

# Photo-Actuation of Liquid Crystalline Elastomer Materials Doped with Visible Absorber Dyes under Quasi-Daylight

**(S- Ⅰ) The ATR-FTIR measurement of the prepared polysiloxane side-chain LCE**

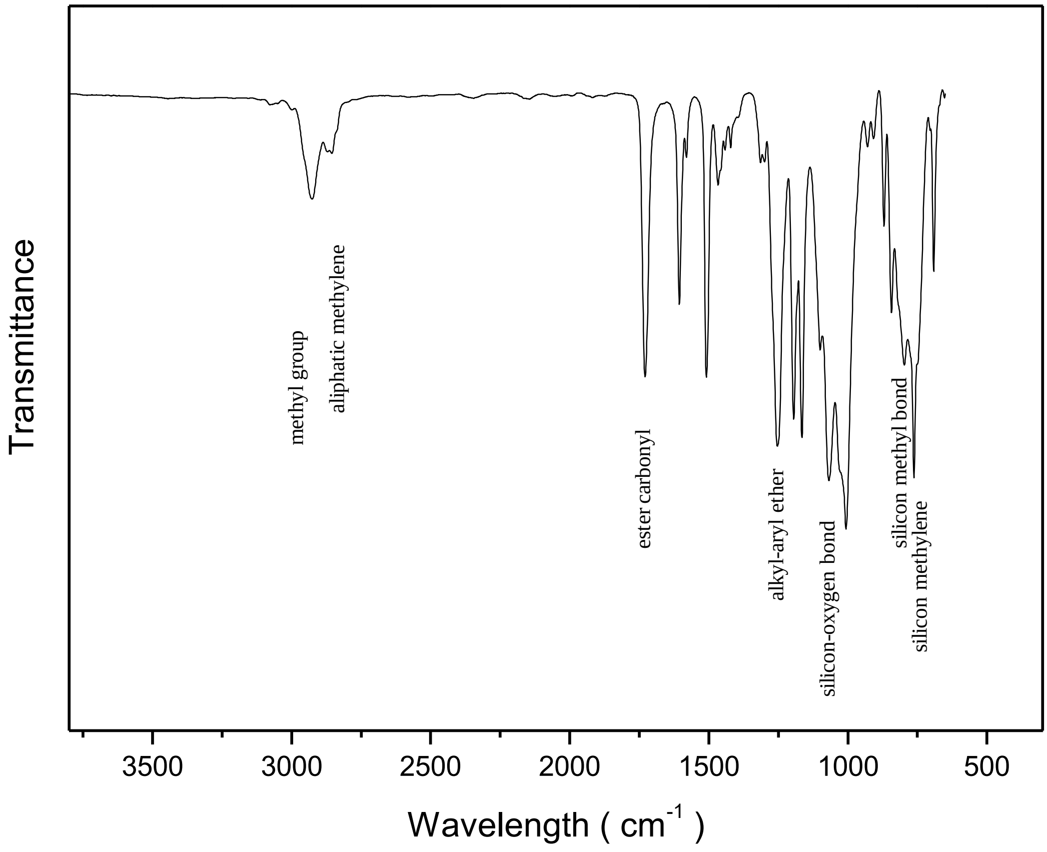


**Figure S1.** ATR-FTIR spectrum of the prepared polysiloxane side-chain LCE.

Attenuated total reflection Fourier transform infrared spectroscopy(ATR-FTIR) on the LCE samples was carried out by a Fourier transform infrared spectrometer (Nicolet 6700, Thermo Electron Corp, USA). Figure S1 shows the measured ATR-FTIR spectrum of the prepared polysiloxane side-chain LCE. The sample exhibited the characteristic peaks of molecular functional groups contained in MBB, 11UB and PMHS:[S1] the peak at about 2942 $cm^{-1}$ was attributed to the vibration of methyl groups (-$CH_3$), the peak at about 2865 $cm^{-1}$ was attributed to the vibration of aliphatic methylene (-$CH_2$-), the peak at about 1730 $cm^{-1}$ was attributed to the ester carbonyl group (-C=O) vibration, the peak at about 1250 $cm^{-1}$ was attributed to the vibration of alkyl-aryl ether (aryl-O-), the peak at around 1070 $cm^{-1}$ was attributed to the vibration of silicon-oxygen bonds (Si-O-Si), the peak at about 795 $cm^{-1}$ was attributed to the vibration of silicon methyl bonds (Si-$CH_3$). But there were no detected characteristic peaks of ethylenic bond (C=C) at about 1620-1680 $cm^{-1}$, alkenyl hydrogen bonds (=$CH_2$) at about 3000-3100 $cm^{-1}$and silicon hydrogen bond (Si-H) at about 2160 $cm^{-1}$, instead the vibration peak of silicon methylene (Si-$CH_2$-) at about 760 $cm^{-1}$ was obviously exhibited. The reason should be that the Si–H bonds in the PMHS reacted with the terminal vinyl groups of MBB molecules and 11UB

molecules and hence generated the silicon methylene (Si-$CH_2$-) bonds. Therefore the ATR-FTIR spectrum confirmed that the LCE network was synthesized through hydrosilation crosslinking reaction as demonstrated in Scheme 2(a).

**(S-Ⅱ) The preparation condition of the polysiloxane side-chain LCE**

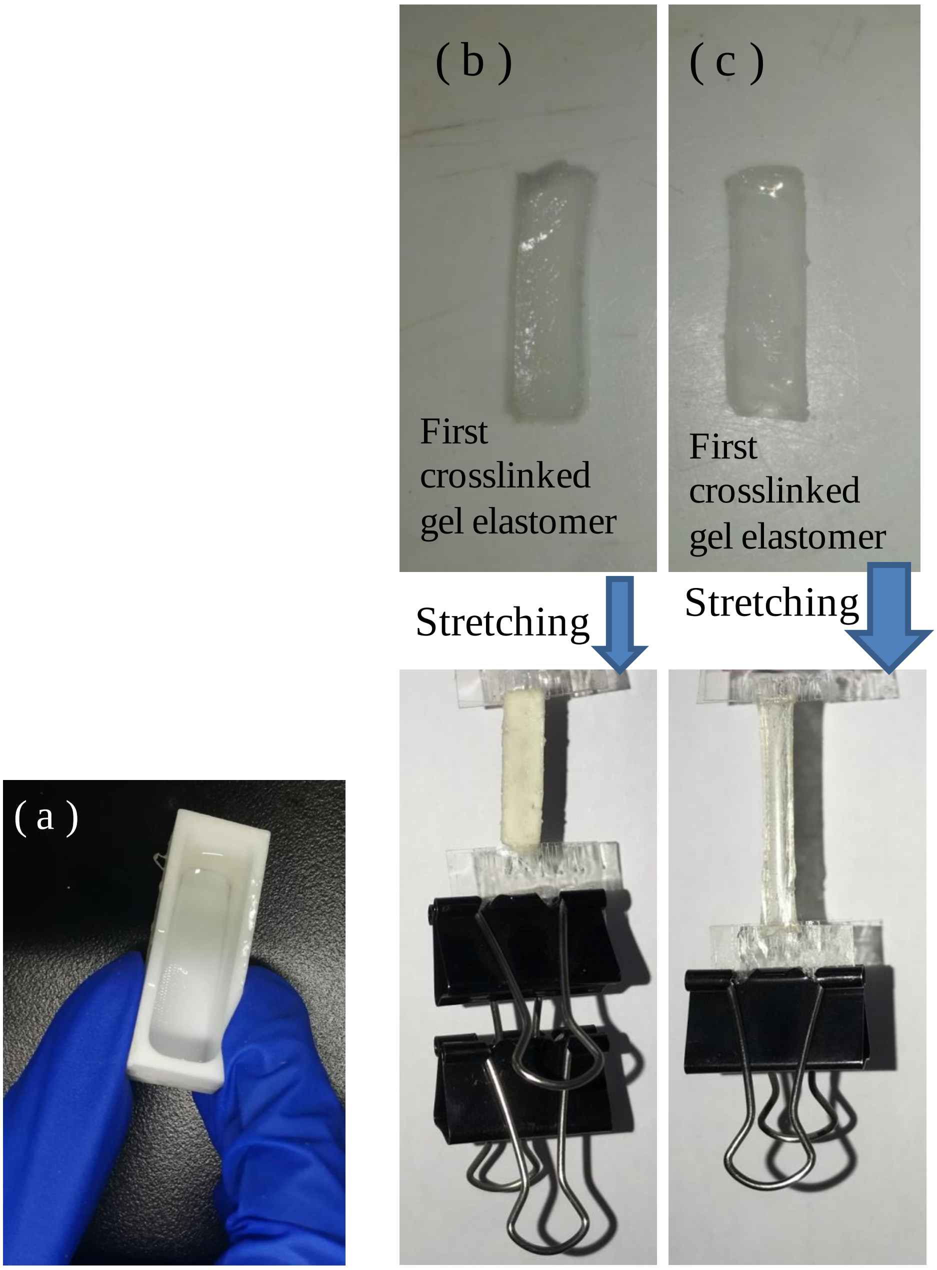


**Figure S2.** (a) The product in the mold was viscous mud state if the reaction time of the first crosslinking step was too short; (b) The gel elastomer generated in the first crosslinking step cannot be effectively stretched if the reaction time of the first crosslinking step was too long; (c) The gel elastomer generated in the first crosslinking step can be normally stretched to form monodomain structure when the reaction condition for the first crosslinking step was optimal.

The preparation of polysiloxane LCE materials are via a sol-gel method cooperated with two-step crosslinking and a stretching process. The matrix networks are generated by the hydrosilylation reaction between the PMHS, MBB and 11UB in the first crosslinking step. Then an external stress is applied to stretch and form a monodomain structure in matrix. After stretching process, the second crosslinking step is used to stabilize the monodomain structure and obtain the nematic LCE material.

The first crosslinking step was critical. Too short reaction time would lead to inadequate reaction degree, the first crosslinking step would not generate a qualified gel elastomer. The product from the first crosslinking step was viscous mud state, and could not be stretched, as shown in Figure S2(a). Too long reaction time for the first crosslinking step would create too high reaction degree, led to high robustness of the generated gel elastomer. The gel elastomer would be hardly extended by stretching process and thus the monodomain structure in matrix would not be formed, as demonstrated in Figure S2(b). Under a suitable reaction time for the first crosslinking step, a qualified gel elastomer could be generated and could be normally extended by stretching process, as demonstrated in Figure S2(c). The monodomain structure in matrix also would be formed during the stretching process. Then through the second crosslinking step, the nematic polysiloxane LCE could be obtained. Herein the second crosslinking step could be proceeded for any time duration which above 12 hours. The performance of the prepared LCE materials could not be influenced by the taken time of the second crosslinking step, as long as the taken time was above 12 hours. In our work, the adopted preparation condition of the LCE materials were the optimum condition through many trying experiments.

**(S-Ⅲ) The photothermal effects of the visible absorber dyes**

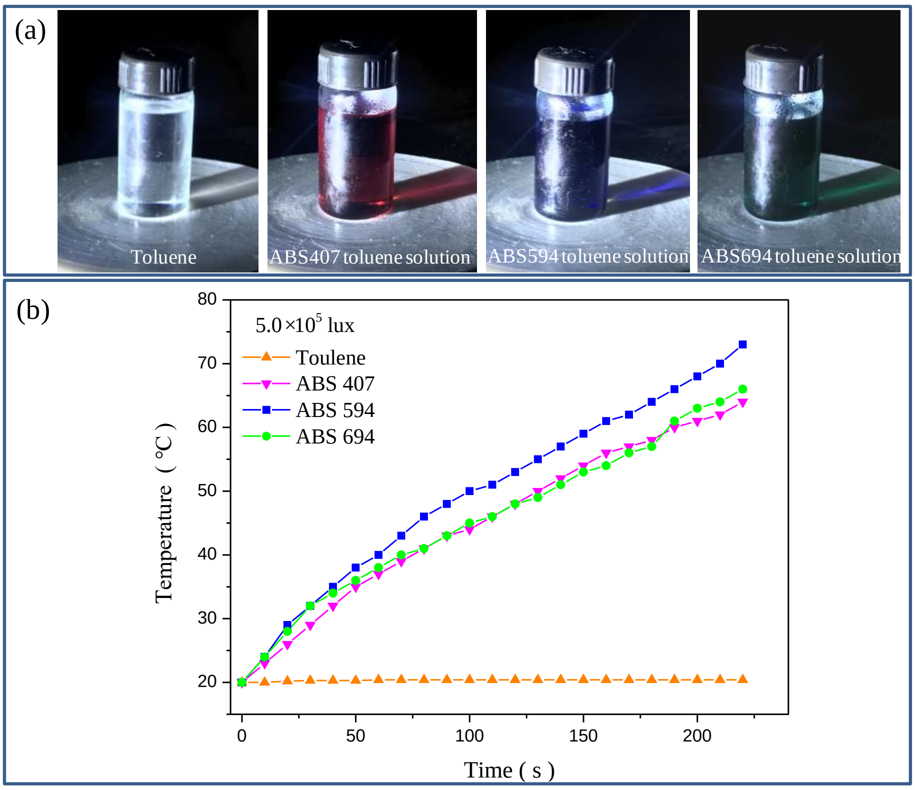


**Figure S3.** (a) The photos of pure toluene solvent, ABS407 dissolved toluene solution, ABS594 dissolved toluene solution and ABS694 dissolved toluene solution. The dye concentration of every solution was 7.5mg/mL; (b) Changes of temperatures of pure toluene solvent, ABS407 toluene solution, ABS594 toluene solution and ABS694 toluene solution versus time under quasi-daylight irradiation with $5.0\times10^5$ lux of intensity.

In order to confirm the photothermal conversion effect of the visible absorber dyes, we measured the temperature changes of the pure toluene solvent and the dye dissolved toluene solutions upon light irradiation. Figure S3(a) shows the toluene solvent, ABS407 toluene solution, ABS594 toluene solution and ABS694 toluene solution. They were filled in glass bottles, the temperature changes of them were measured by using the multilogger thermometer, the measured data were plotted in Figure S3(b). It indicates that when they were irradiated by quasi-daylight irradiation, the

temperature of pure toluene solvent basically did not vary, but the temperatures of the dye dissolved toluene solutions obviously increased as the irradiation time prolonged. The results confirmed that the visible absorber dyes can absorb the light irradiation and converted the photo energy into thermal energy to increase the temperature. The pure toluene is transparent and has no light absorption ability, thus did not generate the photothermal conversion effect.

### (S-Ⅳ) The photo actuation performances of dye doped LCE materials with different thickness

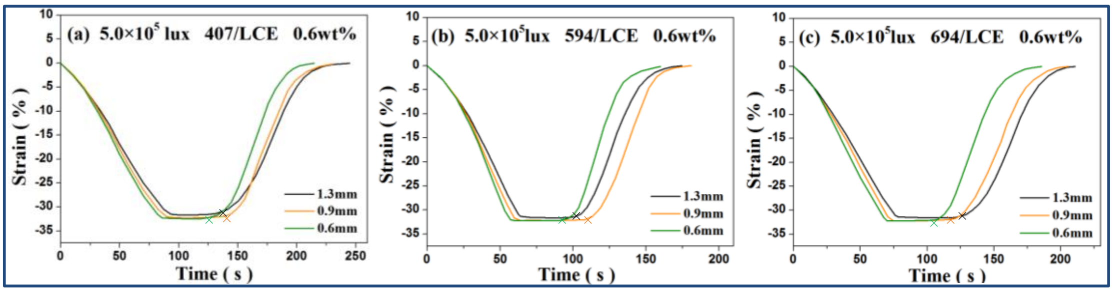


**Figure S4.** Changes in axial strain of every kind dye-doped LCE material (0.6 wt% dye content), with the thickness being 0.6mm, 0.9mm and 1.3mm respective, versus time under quasi-daylight irradiation with $5.0×10^5$ lux of intensity. The mark "×" at every curve signs the time when the light source was turned off.

In order to investigate the influence of LCE film thickness on the photo-actuation performance. In addition to the dye-doped nematic LCE materials with the thickness of 0.6mm which were mainly used in our this work, we additionally prepared three kind dye-doped nematic LCE materials with 0.6 wt% content of visible absorber dye and the thickness being 0.9mm and 1.3mm respective. The axial strains of every kind dye-doped LCE material, with the thickness being 0.6mm, 0.9mm and 1.3mm respective, versus irradiation time are plotted in Figure S4. It indicates that upon the irradiation intensity of $5.0×10^5$ lux, the contraction strains of the three kind dye-doped LCE materials with difference thickness all finally reached the maximum contraction ratio, but the taken irradiation time for reaching final contraction strain slightly increased as the material thickness increased. This revealed that the strain rate in response to light irradiation became slower as the material thickness increased. The reason was that a larger material thickness would increase the resistance of thermal diffusion, resulted in a slower photo-actuation rate of LCE material.

### Reference:

[S1] Larkin, P. J. Infrared and Raman spectroscopy: Principles and spectral interpretation. Elsevier, USA, **2011**
